# Supplementary material for: Simultaneous Determination of 21 Sulfonamides in Poultry Eggs Using Ionic Liquid-Modified Molecularly Imprinted Polymer SPE and UPLC–MS/MS
Source: Molecules. 2022 Aug 4;27(15):4953. doi: 10.3390/molecules27154953 (PMC9370267; doi:10.3390/molecules27154953)

Table S1 Gradient mobile phase for LC

Time（min）	Flow (mL/min)	0.1 % f formatic acid (%)	0.1 % formatic methanol (%)
0	0.3	90	10
1	0.3	90	10
3.5	0.3	75	25
6.5	0.3	70	30
8	0.3	20	80
9	0.3	10	90
9.1	0.3	90	10
10	0.3	90	10

Table S2 The peak time and MRM condition of 21 sulfonamides

Drug	ion pairs (m/z)	cone (V)	CE (eV)
Sulfadiazine	251.0 >92.0	12	24
	251.0 >155.9*		14
Sulfamethoxazole	254.0 >92.1	22	26
	254.0 >155.9*		14
sulfathiazole	256.0 > 92.0	12	24
	256.0 >155.9*		14
Sulfamerazine[b]	265.1 > 92.0	36	26
	265.1 > 156.0*		14
Sulfafurazole[b]	268.0 > 92.0	12	24
	268.0 > 155.9*		12
sulfadimethoxine	311.0 > 92.0	12	28
	311.0 > 155.9*		18
Sulfadoxine	311.1 > 92.0	20	28
	311.1 > 155.9*		16
Sulfamethizole	271.1 > 92.0	12	24
	271.1 > 155.9*		12
Sulfabenzamide[c]	277.0 > 92.0	14	24
	277.0 > 155.9*		10
Sulfisomidine	279.0 > 124.0	12	20
	279.0 > 186.0*		14
Sulfamethazine	279.0 > 92.0	12	28
	279.0 > 186.0*		16
Sulfametoxydiazine	281.0 > 92.0	12	28
	281.0 > 155.9*		16
Sulfamethoxypyridazine	281.0 > 92.0	13	28
	281.0 > 155.9*		16
Sulfamonomethoxine	281.0 > 92.0	12	28
	281.0 > 155.9*		16
Sulfachloropyridazine	285.0 > 92.0	14	26
	285.0 > 156.0*		12
Sulfachloropyrazine[c]	285.0 > 92.0	14	26
	285.0 > 156.0*		12
Sulfaquinoxaline	301.1 > 92.0	12	26
	301.1 > 155.9*		14
Sulfanitran	336.1>156.0*	20	20
	336.1>294.0		10
Sulfaphenazole	315.3>158.2*	9	15
	315.3>160.0		
sulfapyrazole	329.0>156.2*	11	12

Drug	ion pairs (m/z)	cone (V)	CE (eV)
Sulfisoxazole	329.0>294.0	12	10
	268.0 > 92.0		24
	268.0 > 155.9*		12
Sulfadiazine-C6	257.1>162.1	20	15
sulfamethoxazole-C6	259.9>161.9	16	16
sulfathiazole-C6	262.2>162.2	10	19
Sulfisoxazole-C6	273.9>161.9	18	14
Sulfamethizole-C6	277.2>162.1	19	21
Sulfamethoxypyridazine-D3	284.2>156.1	19	28
Sulfisomidine-C6	285.1>186.2	19	21
Sulfamethazine-C6	285.5>162.1	18	16
Sulfametoxydiazine-C6	287.0>162.1	24	14
sulfachloropyridazine-C6	291.2>162.2	24	27
Sulfaquinoxaline-C6	306.8>162.2	13	15
sulfadimethoxine-C6	317.1>162.2	13	25
Sulfaphenazole-C6	321.1>162.1	19	21
sulfapyrazole-C6	335.2>162.2	13	25
Sulfadoxine-C6	314.1>156.1	19	21
sulfamonomethoxine-C6	287.1>162.2	19	28
Sulfanitran-C6	342.1>162.2	19	28

* quantitative ion, quantitative ion used for quantification with isotopically labeled analogue as IS. a, quantitative ion used for quantification with Sulphacetamide -C6 as IS.b,Product ion used for quantification with Sulfamethizole-C6 as IS.c,Product ion used for quantification with Sulfaquinoxaline-C6 as IS.

Figure S1 : The adsorption values(Q value) for SMZ of different MIP

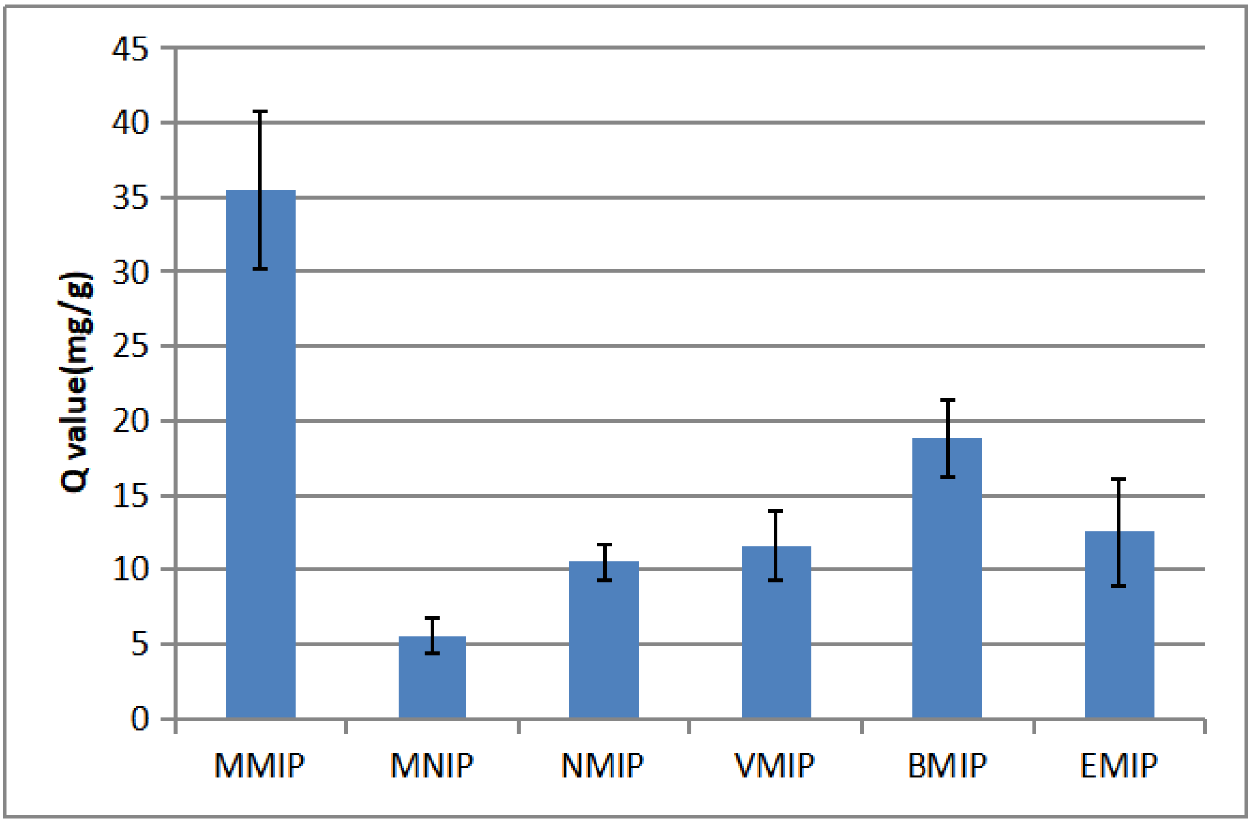

Figure S2 : the FT-IR spectra of MMIP, NMIP, and MNIP

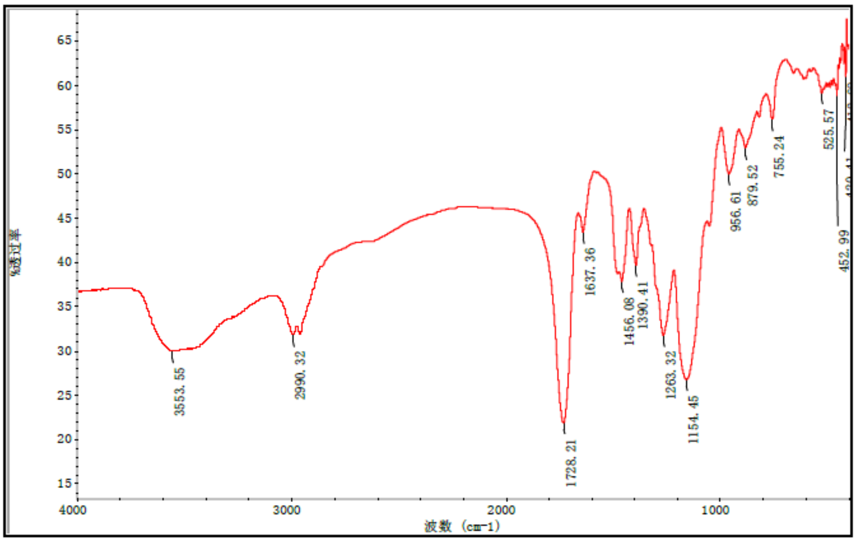

MMIP

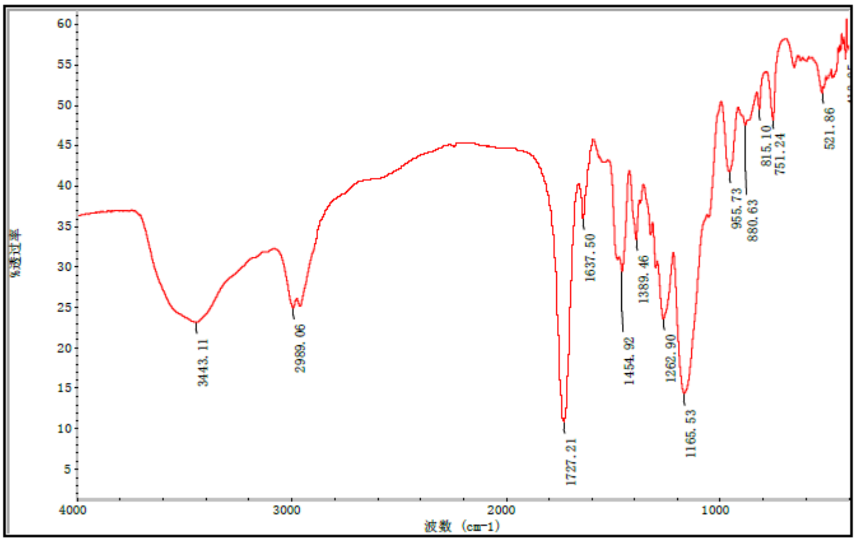

MNIP

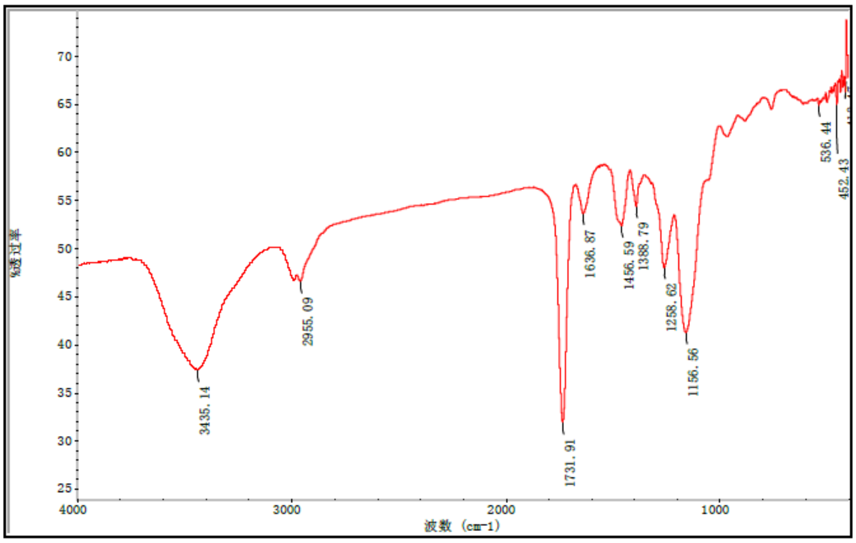

NMIP

Figure S3: Recoveries of different elution

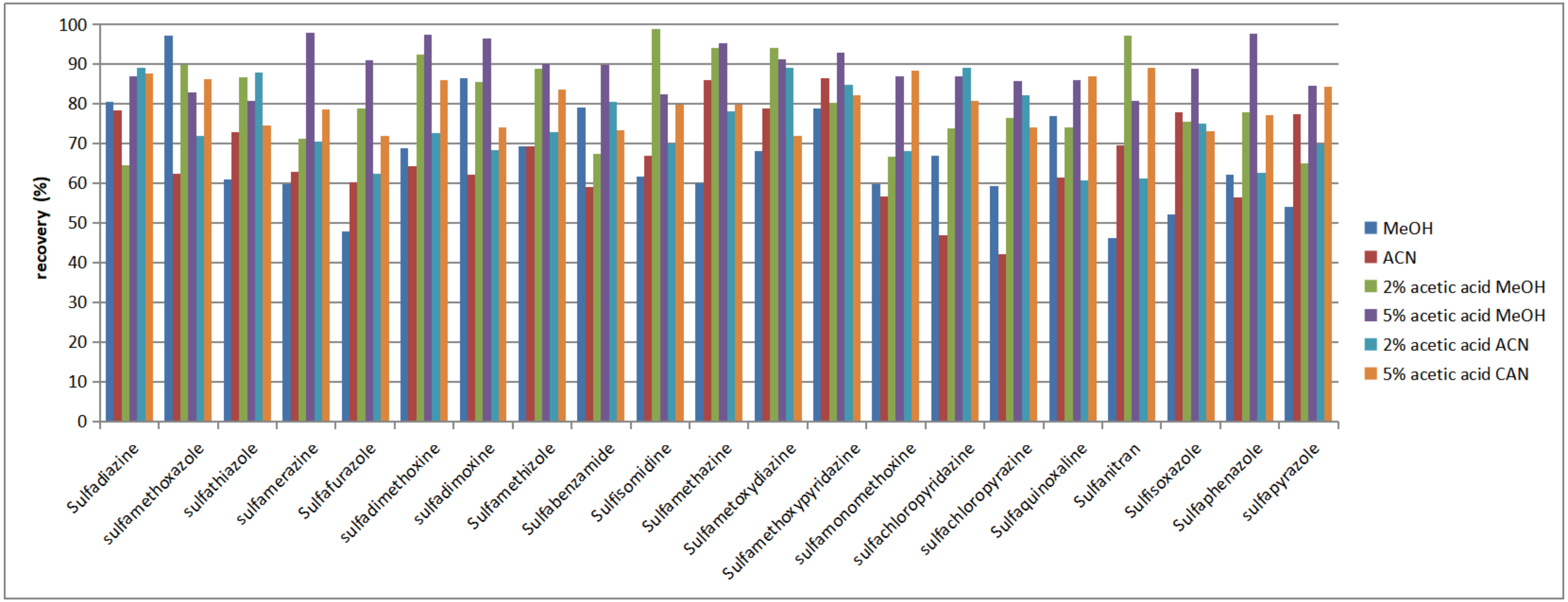

Figure S4: the Reusability of MMIP

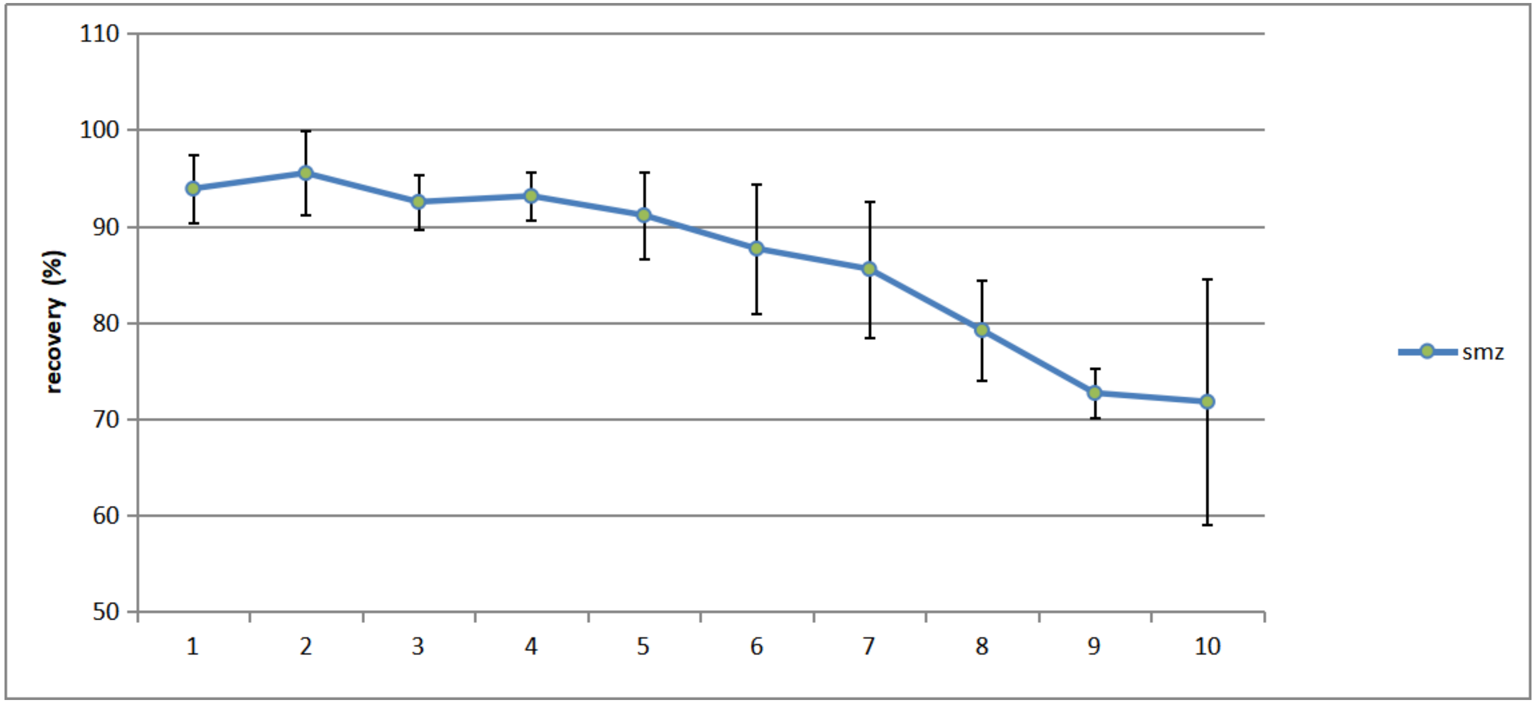

Supplement: Supplementary file 1 [file molecules-27-04953-s001.zip › molecules-1810328-supplementary.pdf]
